# Livestock metabolomics and the livestock metabolome: A systematic review

**DOI:** 10.1371/journal.pone.0177675

**Published:** 2017-05-22

**Authors:** Seyed Ali Goldansaz, An Chi Guo, Tanvir Sajed, Michael A. Steele, Graham S. Plastow, David S. Wishart

**Affiliations:** 1 Department of Agriculture, Food and Nutritional Sciences, University of Alberta, Edmonton, Alberta, Canada; 2 Department of Biological Sciences, University of Alberta, Edmonton, Alberta, Canada; 3 Department of Computing Science, University of Alberta, Edmonton, Alberta, Canada; Wageningen UR Livestock Research, NETHERLANDS

## Abstract

Metabolomics uses advanced analytical chemistry techniques to comprehensively measure large numbers of small molecule metabolites in cells, tissues and biofluids. The ability to rapidly detect and quantify hundreds or even thousands of metabolites within a single sample is helping scientists paint a far more complete picture of system-wide metabolism and biology. Metabolomics is also allowing researchers to focus on measuring the end-products of complex, hard-to-decipher genetic, epigenetic and environmental interactions. As a result, metabolomics has become an increasingly popular “omics” approach to assist with the robust phenotypic characterization of humans, crop plants and model organisms. Indeed, metabolomics is now routinely used in biomedical, nutritional and crop research. It is also being increasingly used in livestock research and livestock monitoring. The purpose of this systematic review is to quantitatively and objectively summarize the current status of livestock metabolomics and to identify emerging trends, preferred technologies and important gaps in the field. In conducting this review we also critically assessed the applications of livestock metabolomics in key areas such as animal health assessment, disease diagnosis, bioproduct characterization and biomarker discovery for highly desirable economic traits (i.e., feed efficiency, growth potential and milk production). A secondary goal of this critical review was to compile data on the known composition of the livestock metabolome (for 5 of the most common livestock species namely cattle, sheep, goats, horses and pigs). These data have been made available through an open access, comprehensive livestock metabolome database (LMDB, available at http://www.lmdb.ca). The LMDB should enable livestock researchers and producers to conduct more targeted metabolomic studies and to identify where further metabolome coverage is needed.

## Introduction

Metabolites are sometimes referred to as the “canaries” of the genome [1]. Just as canaries for coalminers served as sensitive indicators of problems in coal mines, metabolites can be exquisitely sensitive indicators of problems in the genome (as well as the transcriptome or proteome). Metabolites are effectively the end products of complex interactions occurring inside the cell (the genome) and events, exposures or phenomena occurring outside the cell or organism (the environment). As a result, the comprehensive measurement of metabolites (via metabolomics) allows one to determine interactions between genes and the environment. In other words, metabolomics allows researchers to obtain a highly sensitive and more complete description of the phenotype [2; 3]. This metabolic readout of the phenotype is often called the “metabotype” [4]. Recent advances in both analytical chemistry and metabolite data analysis techniques are now making metabolomics far more accessible to a wider range of research disciplines. Indeed, metabolomics is now routinely used in biomedical research (for biomarker discovery and disease mechanism research), food and nutritional analysis, crop characterization and environmental monitoring [5; 6; 7; 8]. As a result, the field of metabolomics has experienced very rapid growth with just two papers published on the subject in 1999 to more than 2400 in 2015.

However, unlike in other areas of agriculture research where metabolomics is widely used in crop trait selection, pesticide monitoring, crop breeding or crop evaluation [9; 10; 11; 12], the application of metabolomics to livestock research is somewhat less widely used or appreciated. This is surprising given the potential of metabolomics to address many important questions in livestock and animal science. In particular, the power of metabolomics to non-invasively detect subtle phenotypic changes, innate phenotypic propensities and dietary responses makes it an ideal tool for livestock research, breeding and assessment [13; 14; 15; 16; 17; 18; 19]. Recently, there have been a number of papers in livestock metabolomics that have generated compelling results showing how metabolomics and metabolite-based phenotyping (metabotyping) can help farmers, veterinarians, livestock researchers and the livestock industry. These include papers demonstrating how metabolomics can be used to predict feed efficiency and residual feed intake (RFI) [20], ascertain disease propensity [21; 22; 23], evaluate dietary responses to different feeds [24; 25], assess carcass merit [26; 27; 28], fertility [29], milk quality [30; 31], determine bioproduct content [32] and ascertain other important economic or breeding traits associated with livestock.

Fast, effective, and quantitative phenotyping is critical for farm trials dealing with animal selection and breeding. Many traditional phenotypic measurements such as those related to animal feed consumption and RFI are expensive, time consuming and require specific recording equipment [20]. Others, such as carcass trait evaluation, may require animal slaughter, which obviously eliminates the potential breeding value of the animal. Similarly for reproductive traits, animals have to reach a stage of maturity and sexual activity to allow measurement of related traits. Metabolomics allows many of these trait measurements to be conducted earlier, more routinely, non-invasively and often at a lower cost than current techniques [33; 4]. However, metabolomics is not without its challenges. Metabolomic experiments must be carefully designed as diet and other variables such as sex, diurnal variations and sampling time can profoundly affect results. Likewise, metabolomic technologies, such as gas chromatography (GC), mass spectrometry (MS) and nuclear magnetic resonance (NMR) spectroscopy are not yet widely available in many livestock research facilities. Furthermore, there continues to be a significant shortage of data resources that could facilitate the interpretation of livestock metabolomic data.

Given the many applications of metabolomics in both the livestock industry and livestock research as well as the diversity of journals in which livestock metabolomics is often published, we felt it was important to conduct a thorough, systematic review of the field. By consolidating the results from diverse journals and different studies into a single review paper, we believed this content would provide a more complete picture of both the strengths and the weaknesses of livestock metabolomics. In conducting this review we sought answers to 4 key questions: 1) What are the most common applications of metabolomics in animal science and where are they trending?, 2) What are the preferred metabolomics technologies in livestock metabolomics and how are they evolving?, 3) What are the most obvious gaps or weaknesses in livestock metabolomics relative to other fields of metabolomics research? and 4) What are the known or measured metabolites for the 5 major livestock species (i.e., bovine, ovine, caprine, equine, and porcine) in different tissues and biofluids? This metabolite compilation, which we have called the livestock metabolome database or LMDB (available at http://www.lmdb.ca), is intended to help lay a more solid foundation in terms of data resources that would make livestock metabolomic studies much easier to perform, analyze and compare. The LMDB catalogues all metabolite compounds that have ever been identified and reported in the 5 livestock species (for multiple biofluids and tissues), along with concentration ranges, compound descriptions, chemical structures, reference NMR and MS spectra and other information associated with each metabolite for both healthy and a variety of abnormal physiological conditions.

## Materials and methods

**Fig 1:**
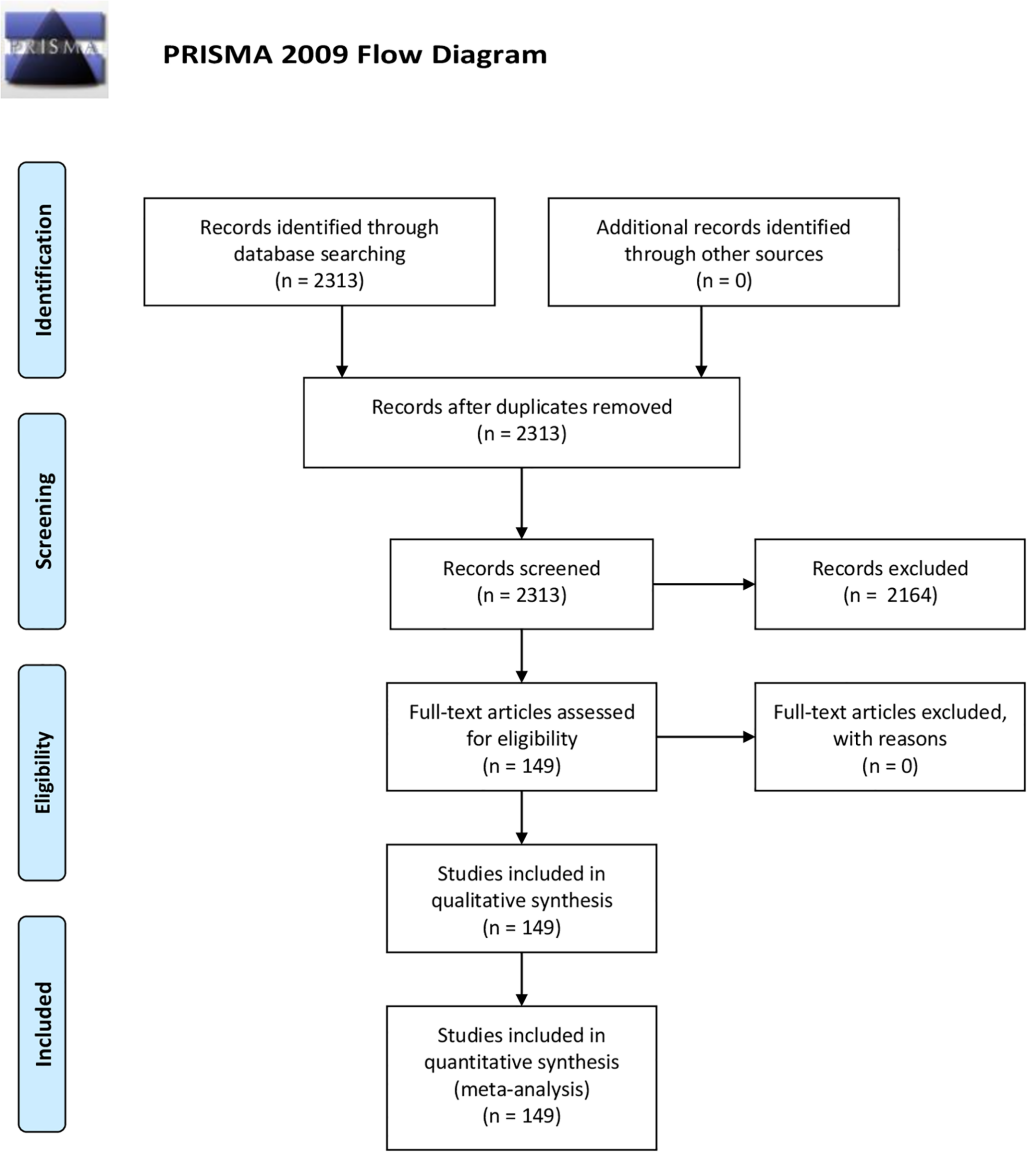
PRISMA diagram. The preferred reporting items for systematic reviews and meta-analysis (PRISMA) flow diagram identifies the total number of articles initially surveyed, the number of articles included and excluded for this systematic review. From: Moher D, Liberati A, Tetzlaff J, Altman DG, The PRISMA Group (2009). Preferred Reporting Items for Systematic Reviews and Meta-Analyses: The PRISMA Statement. PloS Med 6(7): e1000097. doi:10.1371/journal.pmed1000097. **For more information, visit** www.prisma-statement.org.

In compiling this review and assembling the livestock metabolome database, we used a combination of web-accessible data mining tools along with manual curation to survey 2313 peer reviewed journal articles covering the period from 1930 to 2015. From this initial set of articles, we reduced the number further to cover published livestock papers reporting the measurement or characterization of ≥8 metabolites for any of the 5 major livestock species (i.e., bovine, ovine, caprine, equine, and porcine). This reduced the target number of peer-reviewed manuscripts to a total of 149. The livestock species selected for this review were based on their global population, economic impact and use in agricultural systems [34; 35]. Details regarding the keyword selection, search engines and databases, journals and search strategy are given below and summarized in the preferred reporting items for systematic reviews and meta-analysis (PRISMA) checklist (S1 Table) and flow chart (Fig 1).

### Keyword selection

As noted above, this review is focused on 5 main livestock species including cattle, sheep, goats, horses and pigs. Therefore, a combination of keywords was selected to target those specific animals and to identify the associated metabolomics studies. Keywords were divided into 3 main groups: 1) animal species, 2) sample types, and 3) metabolomic methods. Selected keywords for animal species included the name of the species and its various derivatives or synonyms, i.e., bovine, cattle, cow, calf, *Bos taurus*, etc. To target metabolomics papers in animal science, a broad range of metabolomics keywords were identified and used. These included different variations of the term “metabolomics” (such as metabolomics, metabonomics, metabolite profiling, metabolite fingerprint, chemical profile, chemical analysis, chemical composition, etc.) to target publications prior to and after 1999, as well as the names of various analytical platforms (i.e., NMR, mass spectrometry, liquid chromatography, gas chromatography-MS, etc.). Moreover, a wide variety of sample types such as different body fluids (i.e., serum, blood, plasma, urine etc.) and different organs or tissues were selected to further enrich the keyword search.

### Search engines and databases

An initial comparison among many open access search engines showed that most search results are similar regardless of the search engine used. Therefore, Google Scholar (https://scholar.google.ca/) was selected as the primary literature search engine. In addition, a number of agriculture-specific databases such as Agricola and AGRICULTUREnetBASE were also used. Other databases included Scopus, the Web of Science, ScienceDirect (http://www.sciencedirect.com/) and PubMed (http://www.ncbi.nlm.nih.gov/pubmed). Settings for all search engines and databases were adjusted to increase search efficiency and filter irrelevant results.

### Search methods and selection criteria

Different keywords were combined to target metabolomics papers in the field of animal science. For example, “cattle”, “cattle serum” or “cattle milk” was accompanied with “metabolomics”, “chemical composition” or “metabolite profiling”. Consequently, each combination of the keywords in the search engines generated a long list of results. These included various types of publications (full papers or abstracts) that contained any or all of the used keywords. A manual review was performed on all retrieved publications. Typically, the first 3–5 pages of the search results from the aforementioned search engines were manually reviewed to select for articles of interest. Among the papers identified as worth pursuing, research papers, abstracts or textbooks that showed relevance in their title or abstract were selected. In addition to papers reporting experimental results, review articles that included specific metabolite data sets were also selected. Among the selected manuscripts, only those papers that reported ≥8 metabolites were chosen for this review. The threshold of 8 or more as the minimum number of metabolites was based on a *post hoc* analysis of the retrieved papers and the need to optimize both metabolite coverage and the time devoted to manual analysis. We also determined that this selection cut-off allowed us to cover most, if not all, of metabolites reported in papers with <8 metabolites. Based on these criteria, a total number of 149 manuscripts covering all 5 animal categories were selected for this review. Selected publications were carefully read to extract and annotate a set of 10 pieces of information including: 1) metabolite names; 2) tissue or biofluid origin; 3) quantified values (concentration) if any; 4) experimental conditions; 5) animal breed; 6) sample size; 7) analytical platform; 8) field of research, 9) physiological condition (disease or state of health), and 10) Pubmed/DOI references.

### Compilation of the livestock metabolome database

In compiling the data for this livestock metabolome database or LMDB (http://www.lmdb.ca), all reported concentrations were transformed into a standardized concentration unit (micromolar; μM) and each entry was associated with an abbreviated description of the experimental context, the sample type, and the methodologies used for the metabolomic analyses. In identifying a metabolite for inclusion in this study the compound had to: 1) have a molecular weight <1500 daltons; 2) it could not be a peptide, protein or oligonucleotide; 3) it had to correspond to a reasonably unique chemical entity (triglycerides and amino acids are not unique chemical entities, but LysoPC-16:2 is sufficiently unique) and 4) it had to be identified with a structurally interpretable name. This literature-based effort generated 1070 metabolites from 149 peer-reviewed papers, abstracts or textbooks. Metabolites extracted from these manuscripts were systematically categorized into the LMDB. Nearly all metabolites extracted were linked to a standard Human Metabolome Database (HMDB) identifier [36; 37; 38] which provides a freely-accessible comprehensive description of each metabolite. A brief description of experimental data for each metabolite was also extracted from the articles and included in the database including information on the analytical platform, experimental conditions and field of research. A PubMed and/or DOI id was also associated with each metabolite to provide a link to the article reporting that metabolite. Additional data on each metabolite, including structure, synonyms, chemical classifications, physicochemical data, reference NMR, GC-MS or LC-MS spectra and links to other databases were obtained through an in-house annotation tool called DataWrangler. All of this information was used to construct the on-line version of the LMDB (http://www.lmbd.ca). The LMDB was prepared using a Ruby-on-Rails [39] framework, modeled after other on-line species-specific metabolomic databases prepared in our laboratory. Details regarding their construction, required operating systems, browser compatibility and hardware requirements can be found elsewhere [36; 40; 41].

## Results and discussion

### Growth and trends in livestock metabolomics research

**Fig 2:**
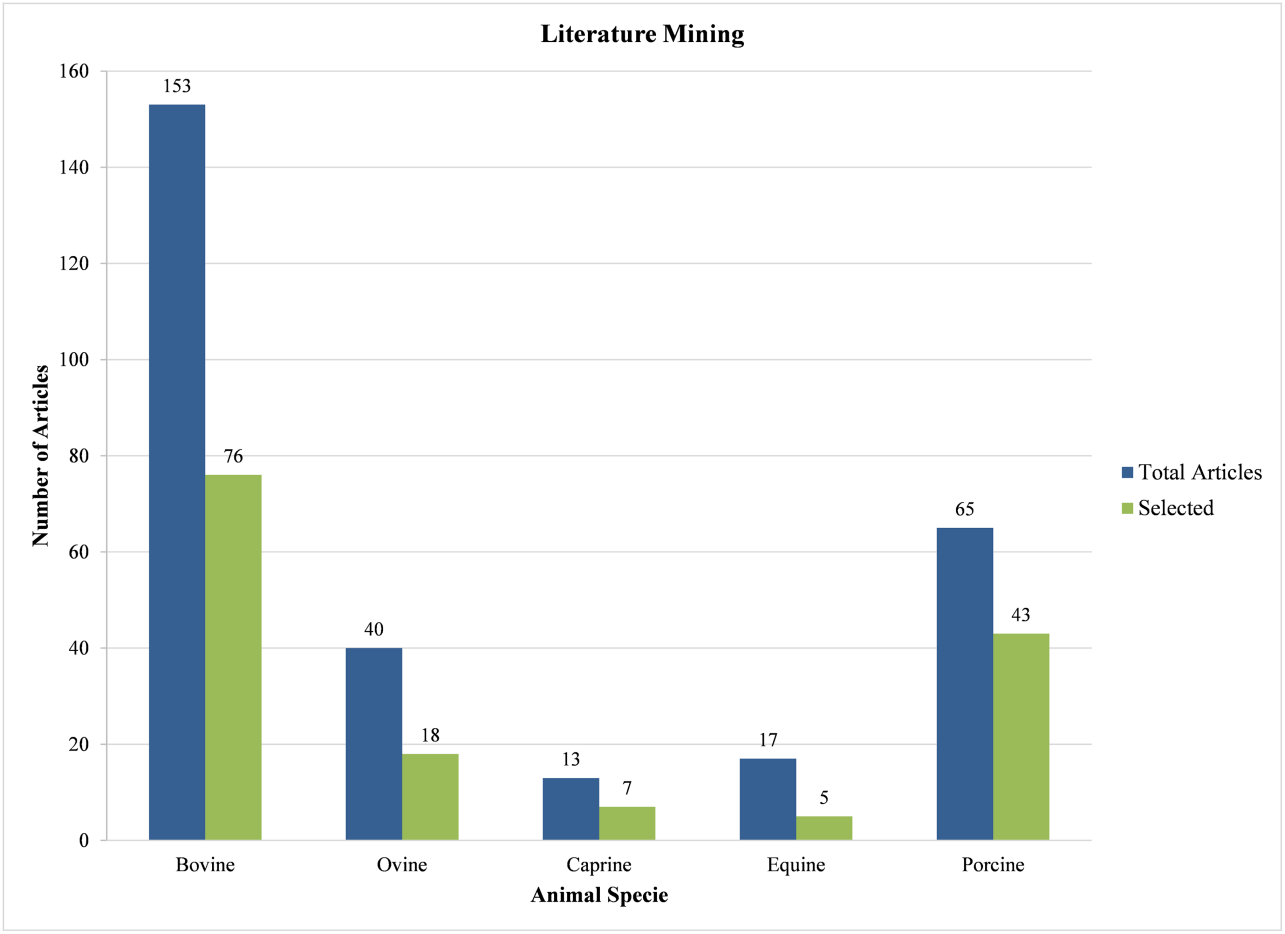
Literature mining. Total number of livestock metabolomics articles considering only articles that reported ≥8 metabolites resulted in selection of 149 manuscripts for this review.

Based on the data collected from our literature survey, it is clear that the majority of metabolomics studies among all livestock categories have been conducted in cattle (Fig 2) with a total of 76 articles (50% of the selected articles) focusing on various fields of bovine research and assessment. Metabolomics studies on pigs and sheep came second and third with 28% and 12% of the selected articles, respectively. The least studied group were horses with only 5 (3%) reported equine metabolomic studies. As might be expected, most livestock metabolomic studies focused on issues related to animal health, nutrition and production (65%). These studies are obviously useful for characterizing bioproduct quality, identifying biomarkers or understanding animal responses to different stressors. However, we were surprised to see relatively few efforts focused on metabolomic characterization of healthy animals with the aim of identifying baseline values for different metabolites in different biofluids or tissues. In fact, only 16 studies (10%) of this kind were reported. These “referential surveys” are foundational and are often needed before biomarker studies could/should be undertaken or fully understood.

As noted earlier, most metabolomic studies of cows, sheep, goats and pigs appear to be directed towards disease detection, production and bioproduct assessment, feed efficiency determination and reproduction. In contrast, the primary focus for equine metabolomics has been on drug discovery and doping detection, specifically for thoroughbred horses [42]. Given the large sums of money directed to horse racing, this is not unexpected. However, compared to the widespread applications of metabolomics in other livestock species for other purposes, it is clear that equine metabolomics is being under-utilized. Certainly, equine metabolomics could be used to select more desirable traits and higher value or higher performing animals, similar to what is being done for bovine metabolomics. Likewise, metabolomics could serve as a diagnostic or prognostic tool for improving equine health and disease resilience (as it has for essentially all other livestock species).

Temporal categorization of all 149 published studies showed that the majority of livestock metabolomics papers were published after 1999. Less than 9% (13 articles) of the selected papers were published prior to 1999 while, ~91% (136 articles) of the papers were published thereafter. The earliest paper in our collection dates from 1930. It is noteworthy that the term “metabolomics” was not coined until 1998 [43; 44] therefore, metabolomics studies prior to this date had to be identified using other keywords such as “chemical composition”, “biochemical profiling”, etc. Based on our observations, it is clear that interest in livestock metabolomics is growing rapidly, especially over the last couple of years. Our data indicates that from 2000–2010 just 29 articles (19%) were published in this field, while from 2011–2015 a total of 107 (72%) articles were published. In terms of percentage growth, the most rapidly expanding subfield appears to be caprine and equine metabolomics with a growth rate of 100% over the past 5 years. In terms of overall growth, the most significant changes were in bovine metabolomics with the number of papers growing from just 10 prior to 1999 to 49 in 2011–2015. The most recent additions to the field of livestock metabolomics are studies focused on goats (starting in 2014) and horses (starting in 2007).

### Trends and gaps in livestock metabolomics applications

We found that livestock metabolomics studies can be categorized in 7 main areas (Table 1). These include animal health, animal nutrition, animal production, animal reproduction, animal physiology (mainly analysis of different biofluids), animal products (products originating from livestock such as milk, meat, yogurt, etc.), and human health (livestock models used for human health studies). This general categorization was based on a *post hoc* analysis of the types of articles where we manually assessed article keywords, subject headings, journal titles and the general focus of each article. Most of these categorizations (such as animal reproduction, human health and animal health) were relatively simple to make. For instance, the category “animal reproduction” obviously refers to articles using metabolomics to study reproduction in livestock. Likewise, the category “animal health” refers to articles using metabolomics to study livestock health or disease while “human health” refers to application of metabolomics to study human disease using various livestock models. Other categories proved to be somewhat more ambiguous. For instance, the field of “animal products” typically contains metabolomics investigations related to food, nutrition and human consumption of animal products, such as meat and cheese. On the other hand, “animal production” is focused on investigating the associated biochemical profile with each animal product. In some cases, we had to be fairly strict with our definitions. For instance, we limited “animal physiology” to include only those articles focused on analyzing various biofluids or characterizing the metabolite composition of specific biofluids, organs and tissues.

**Table 1:** Categorical comparison.

	Bovine	Ovine	Caprine	Equine	Porcine
**Animal Health**	30	6	2	4	10
**Animal Nutrition**	10	6	5	0	14
**Animal Production**	22	3	2	2	11
**Animal Reproduction**	2	1	0	0	3
**Human Health**	6	4	2	0	14
**Animal Products**	16	1	0	0	2
**Animal Physiology**	13	2	0	1	0

Selected livestock metabolomics articles of 5 major livestock species were categorized based on the area of research, i.e., animal health, animal nutrition, animal production, animal reproduction, human health, animal physiology and animal products. It is noteworthy that articles in the area of human health mainly reflected animal models being used to study human related health issues.

Among the seven different categories, animal health (52) and animal production (40) had the most metabolomics articles published for the largest number of animal groups (Table 1). However, this varied depending on the livestock species being studied. In human health research, porcine metabolomic studies covered the majority of articles (14 articles) compared to all other livestock categories. This is not unexpected, given the comparable physiology of pigs to that of humans [45]. In the category of animal products, bovine-based studies had the most articles published (16 articles) relative to all other groups. Some of the more interesting applications of metabolomics found in our survey include the use of metabolomics for quality control of animal products [46; 47], evaluating nutritional value and impact of various feed sources on animal health and products [25], investigating disease biology by using animal models of human disease [48; 49], investigation of potential metabolite biomarkers of animal disease [22; 23], assessment of production traits [50; 51], reproductive performance [29], and general metabolome characterization [52; 53].

In terms of gaps in the existing literature, it is perhaps most useful to use bovine metabolomic studies as the “gold standard” by which to compare other livestock species. While metabolomics is routinely being used to understand the biology or diagnose a few common bovine production diseases (including acidosis, mastitis, milk fever) we found no metabolomic studies looking at common diseases in sheep or goats (such as brucellosis, campylobacteriosis, pneumonia, Q fever), in horses (equine flu, equine herpes, equine sleeping sickness, anemia, laminitis, azoturia), or in pigs (respiratory diseases, swine dysentery, parvovirus). Indeed, we found only 22 metabolomic studies focused on the health of sheep, goats, pigs and horses, compared to 30 metabolomic studies for cattle alone. Of these 22 non-bovine studies, most were focused on metabolic, growth and neurodegenerative disorders.

Livestock metabolomics studies also appear to be missing a number of opportunities currently being pursued in human biomedical research. One of particular note is the use of metabolomics to predict (as opposed to diagnose) or detect subclinical forms of disease. While disease diagnosis is useful, often it is too late or too costly to perform useful veterinary interventions. Detecting diseases before they manifest or predicting them before they occur allows inexpensive prophylactic or preventative measures to be taken. In human metabolomic studies, the identification of disease prediction biomarkers is becoming increasingly common [54; 55; 56; 57; 58]. This is because metabolic changes appear to precede significant physiological changes, possibly because metabolites play an important signalling role to activate later stage (i.e. symptomatic) physiological responses [59; 60]. However, we could only find 2 papers (limited to cattle) that focused on disease diagnosis/prognosis or (sub)clinical detection of diseases [21; 61]. A similar approach could also be used towards the prediction of later-life production traits on the basis of early-life metabolic fingerprints. This, too, is an area of interest in the field of human metabolomics, where later-life health is being predicted on the basis of early-life metabolic fingerprints [62; 63; 64]. Obviously the reliable prediction of economically important traits is an important tool for livestock management and strategic planning.

Metabolomics is already being used in the evaluation and/or prediction of production traits such as residual feed intake (RFI), carcass merit, reproductive performance and metabolic disorders for cattle. However, there is a surprising dearth of similar studies regarding evaluation or prediction of production traits for sheep, goats and pigs. Metabolomics potentially offers a unique opportunity for indirect, inexpensive marker-assisted measurement of these economical traits. This can be achieved through non-invasive sample collection of readily accessible biofluids such as blood, urine, milk and saliva. In most cases, the standard measurement or prediction of some traits such as RFI and carcass merit requires labour intensive, invasive, costly and time consuming measurements [65]. Metabolomic studies regarding the prediction of RFI in beef cattle have already been very promising with a reported initial prediction accuracy of 95% [20; 66]. Metabolomic data, when coupled with genomic data, appear to increase the accuracy of trait prediction [67]. This combination potentially allows one to screen for individual animals with superior traits that could be used for breeding stock. Given the positive results already seen for cattle, the application of these metabolomic concepts to other livestock species is certainly worth investigating. Overall it appears that there is still a considerable body of useful metabolomic work that could be pursued with most other livestock species by simply applying or extending what has already been done in bovine metabolomics.

### Trends and gaps in sample size

Nearly 50% of the selected articles for all animal species used ≤30 animals or samples (from an even smaller number of animals) to conduct their metabolomics analysis. Other sample size categories shown in Table 2 account for ~10% of the peer-reviewed livestock metabolomics literature. The maximum number of samples reported from the selected papers were: 1587 (bovine), 163 (ovine), 80 (caprine), 36 (equine), and 506 (porcine). It is noteworthy that sample size does not always reflect the total number of animals used in the study. For instance, longitudinal studies typically collect multiple samples from a relatively small number of animals over an extended period of time. Relative to many reported human metabolomic studies [55] or rodent model studies [68] the number of samples and the number of subjects (i.e. animals) used in most livestock metabolomics studies is generally quite small. Indeed, many human and rodent model studies routinely measure 100s to 1000s of samples. This difference in sample size likely reflects the relatively high cost of performing large animal studies as well as the somewhat limited funding available to agriculture research relative to medical research.

**Table 2:** Sample size.

	Bovine	Ovine	Caprine	Equine	Porcine
**≤30**	25	6	4	5	30
**31–50**	12	2	0	0	7
**51–100**	9	4	3	0	1
**>100**	16	2	0	0	2
**Undetermined**	13	3	0	0	3

Sample size reported in livestock metabolomics papers were divided into 5 categories with papers using ≤30 samples, 31–50, 51–100, or those that have not mentioned the number of samples used in the analysis.

However, it is important to note that the smaller sample sizes in livestock metabolomics also mean that statistical significance and “power” of the published results is also somewhat less than many human-subject or model organism studies. This represents a significant gap for livestock metabolomics and requires either study sizes to be increased or more effort being directed to conducting validation studies on similar-to-largely sized cohorts for confirmation previously reported results. Indeed, we found only one bovine metabolomic study reporting either independent cross validation (using a different animal cohort) or independent follow-up validation of any newly identified biomarkers or interesting metabolite findings [20]. On the other hand, follow-up validation studies are becoming routine in human metabolomic studies [69; 70; 71]. Clearly, this is a gap in livestock metabolomics that must be filled if metabolomic findings are going to be translated to practical pen-side or on-farm applications.

Another consistent problem detected in the published livestock metabolomics literature is incomplete reporting. We found that 13% of all published livestock metabolomics papers did not report the number of samples used in their research. Providing information on sample size is an essential scientific measurement and reflects on the quality and reliability of published papers. Failure to report sample sizes along with failure to provide information on the numbers of animals or animal replicates indicates a major flaw in manuscript preparation and scientific work.

### Trends and gaps in biological sample types

**Fig 3:**
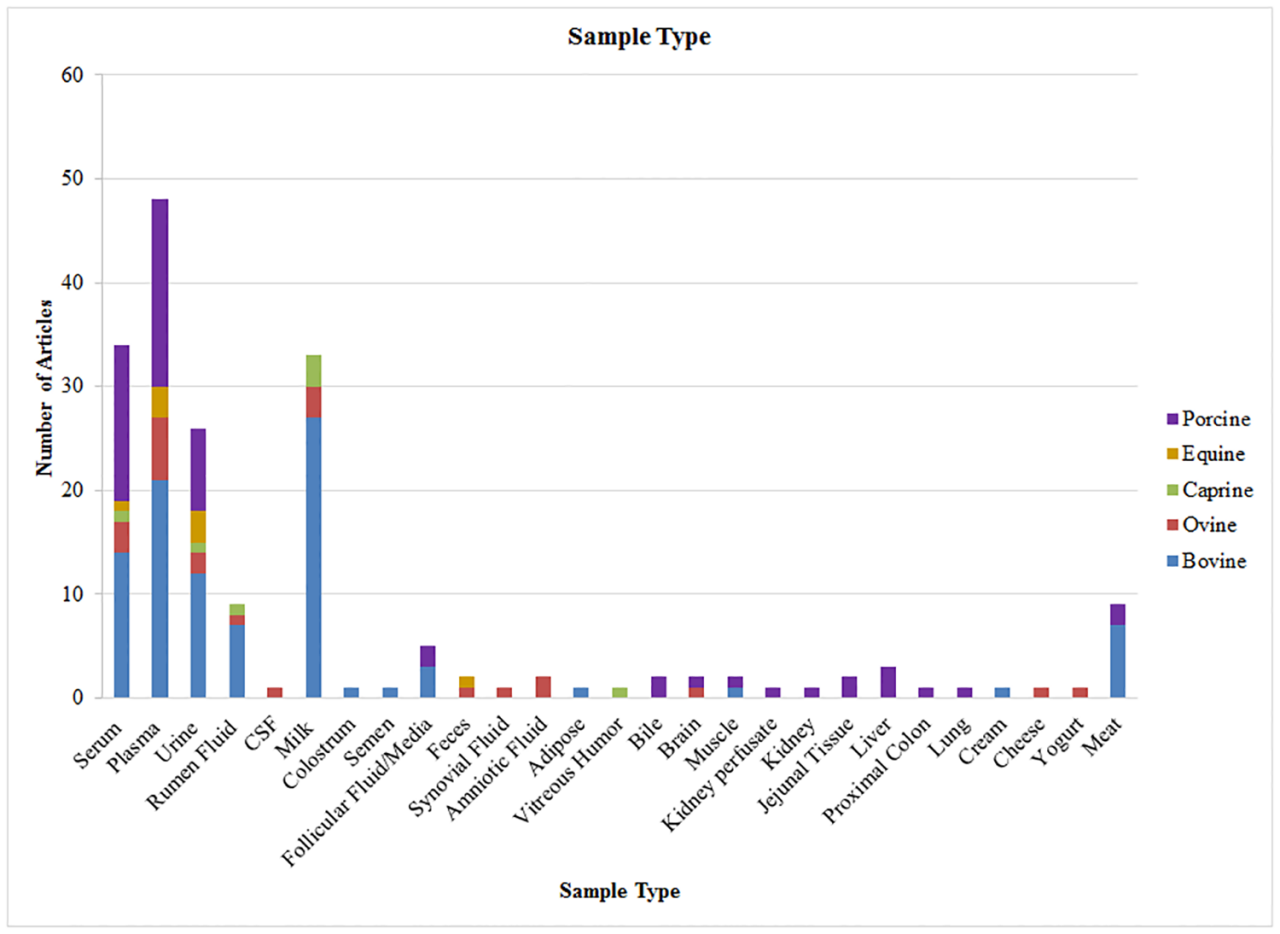
Sample types. Different varieties of samples and animal products have been analyzed in livestock metabolomics studies.

As can be seen in Fig 3 and Table 3, a total of 30 different sample types have been used for livestock metabolomics analyses. The most commonly used sample types include milk, plasma, serum, urine and ruminal fluid. These biofluids account for 78% of the total sample types reported. Milk and plasma are the most commonly used samples in bovine metabolomics manuscripts. Among all other animal groups, plasma was the most widely examined sample type (Table 3), reflecting perhaps the ease of collection but also its potential utility as a proxy reporter for all of the organs in the body [72]. Some of the least frequently used samples include cerebrospinal fluid, colostrum, semen, adipose tissue, kidney and kidney perfusate, feces, amniotic fluid, bile and liver (Table 3). The relatively low number of papers reporting data on tissue metabolomics likely reflects the challenges and costs of animal culling especially for larger livestock, sample collection, and the need to rapidly perform metabolic quenching via liquid nitrogen (immediately after surgery or necropsy) to obtain useful tissue samples for metabolite analysis [73; 74].

**Table 3:** Sample types.

	Bovine	Ovine	Caprine	Equine	Porcine
**Adipose**	1	0	0	0	0
**Amniotic Fluid**	0	2	0	0	0
**Bile**	0	0	0	0	2
**Brain**	0	1	0	0	1
**Cerebral-Spinal Fluid**	0	1	0	0	0
**Cheese**	0	1	0	0	0
**Colostrum**	1	0	0	0	0
**Cream**	1	0	0	0	0
**Feces**	0	1	0	1	0
**Follicular Fluid/Media**	3	0	0	0	2
**Jejunal Tissue**	0	0	0	0	2
**Kidney**	0	0	0	0	1
**Kidney Perfusate**	0	0	0	0	1
**Liver**	0	0	0	0	3
**Lung**	0	0	0	0	1
**Meat**	7	0	0	0	2
**Milk**	27	3	3	0	0
**Muscle**	1	0	0	0	1
**Plasma**	21	6	0	3	18
**Proximal Colon**	0	0	0	0	1
**Rumen Fluid**	7	1	1	0	0
**Semen**	1	0	0	0	0
**Serum**	14	3	1	1	15
**Synovial Fluid**	0	1	0	0	0
**Urine**	12	2	1	3	8
**Vitreous Humor**	0	0	1	0	0
**Yogurt**	0	1	0	0	0

Different varieties of samples have been used in livestock metabolomics analyses as identified by the number of published articles per sample per livestock specie.

While studies on bovine milk are quite prevalent, there are essentially very few studies on sheep or goat milk (Table 3). Given the importance of goat and sheep milk in the global agrifood economy, it is surprising that only a total of 6 papers have been published on goat/sheep milk metabolites. One notable study, however, is that of Park and colleagues [75] who used LC-MS to identify/quantify 82 metabolites in sheep and goat milk. This paper reports a number of other macronutrient milk constituents including fat, protein, minerals and vitamins. In another more recent study, the effect of a specific grazing patterns and their associated dietary effect on goat milk was evaluated [76]. These authors used GC-MS techniques to identify and quantify 25 milk metabolites.

Similar trends are also seen in other biofluid or sample types, with bovine samples or bovine-related papers dominating. For instance, there are a number of metabolomic studies on bovine ruminal fluid, plasma and urine, but very few studies on these biofluids for sheep, goat, horses or pigs (43 for all 4 species and 3 sample types). Likewise, metabolomics studies on colostrum and semen are limited to cattle only with one study each. Interestingly, some of the less-frequently used sample types such as cerebrospinal fluid, synovial fluid, amniotic fluid, bile and vitreous humor are limited to the less frequently studied livestock species (sheep, goat and pig). What is also quite striking is the dearth of fecal metabolomic studies among all livestock species (Table 3). With the growing interest in the microbiome and the clear role that gut (and rumen) microflora play in animal health, we were surprised by the complete absence of metabolomic papers on bovine fecal samples.

Given the importance of beef, sheep and goat meat, it is also surprising to see how little metabolomic data has been collected on meat samples. Indeed, only a total of 9 papers provided data on relatively small number (140) of meat metabolites. The most comprehensive meat metabolomics study was reported by Castejón et al. [32]. These authors profiled meat exudate using NMR to explore the effect of storage time on metabolite composition. They reported a total of 60 different metabolites. Overall, these data suggest that the livestock metabolomic literature is characterized by a significant under-representation of some important sample types, including milk, meat, fecal/rumen, semen samples and cerebrospinal fluid. These “gaps” in our knowledge and “gaps” in the published literature represent clear opportunities for livestock researchers to pursue.

With regards to the number of metabolites detected, quantified and/or reported among the different sample types, we found that the broadest level of coverage was for milk, plasma and serum (Table 4). Ruminal fluid, urine, feces and meat samples had slightly lower levels of coverage while the rest of the sample types reported in Fig 3 typically report <60 metabolites each. It is instructive to compare these livestock metabolite numbers to data reported for human metabolites identified in similar kinds of sample types. For instance, the most comprehensive human milk metabolomics paper reports just 129 identified metabolites [77], which is >3X lower than what has been reported in the livestock milk. The total number of metabolites reported for plasma/serum in humans is 4229 [72], which is significantly more than what is reported for livestock plasma/serum (with 759). Likewise, the total number of human urine metabolites has been reported to be 445 [2], which is more than twice that found in the urine of livestock species. Given their genomic similarity, our expectation is that the number of metabolites measurable in livestock for each of the biofluids should be comparable to the number of metabolites measured in humans. Currently, the Human Metabolome database recognized as the most comprehensive metabolomics database contains >40,000 metabolites derived from various human biosamples [78]. As a result, this suggests there is still a significant gap to be filled with regard to the depth and breadth of metabolome characterization in livestock.

**Table 4:** Metabolite coverage.

	Milk	Plasma	Serum	Ruminal Fluid	Urine	Feces	Meat
**Number of Metabolites**	422	408	351	248	177	158	75

The number of metabolites detected, quantified and/or reported among the commonly used sample types in the livestock metabolomics publications up to 2016 (counting publications that reported >8 metabolites).

### Trends and gaps in analytical instrumentation and methodologies

**Fig 4:**
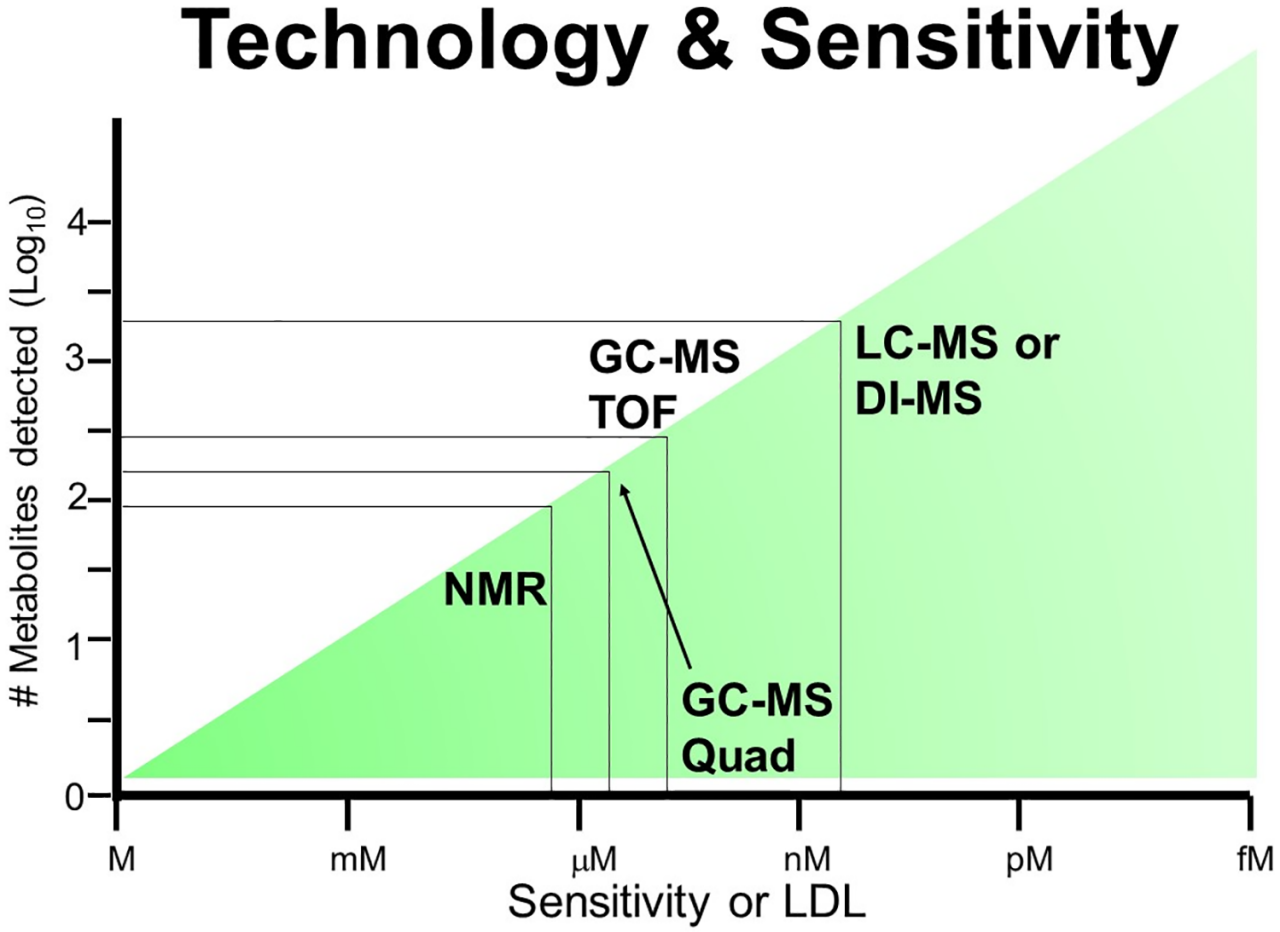
Relative sensitivity of metabolomics platforms. Nuclear magnetic resonance (NMR), gas chromatography-mass spectrometry (GC-MS), and liquid chromatography (LC)-MS are the commonly used metabolomics platforms with varying detection limits.

Metabolomics uses a wide variety of analytical instruments that vary in terms of their sensitivity and breadth of coverage. Nuclear magnetic resonance (NMR) continues to be among the most commonly used analytical platforms in metabolomics [79]. It is often chosen for its reliability and utility in absolute quantitation however, NMR is relatively insensitive and is limited to measuring substances in micromolar to millimolar (μM-mM) concentrations (Fig 4). Mass spectrometry (MS) platforms (especially LC-ESI-MS) can detect metabolites at nanomolar (nM) to picomolar (pM) concentrations, allowing a much higher number of metabolites to be detected. However, MS instruments are prone to frequent breakdowns and, relative to NMR, it is often difficult to quantify chemical concentrations via MS techniques. Gas chromatography-MS (GC-MS) is less sensitive than liquid chromatography (LC)-MS, but is generally more robust and more reproducible. As a result, GC-MS can sometimes be used to identify and quantify the metabolome with higher precision and reproducibility than either NMR or LC-MS.

Each of the 149 livestock metabolomics papers was carefully analyzed to identify which analytical platforms (NMR, LC-MS, GC-MS) were used more frequently to conduct metabolomic analyses. In certain studies, more than one platform was used so, we simply counted the frequency that each technique or technology was used in each study. Interestingly, the most commonly used metabolomics platform for all animal categories is NMR spectroscopy, accounting for 28% of all livestock metabolomics studies. Following closely behind NMR, in terms of frequency, is LC-MS with 25% of all studies using this analytical platform. It is noteworthy that the LC-MS category includes ultra performance liquid chromatography (UPLC)-MS, high-performance liquid chromatography (HPLC)-MS, and direct flow injection (DFI)-MS. Gas chromatography-MS is the third most prevalent (15%) analytical platform used in livestock metabolomics studies. The more limited use of GC-MS is typical of other metabolomic disciplines as well.

Other, less conventional or more targeted, methodologies account for the remaining 27% of the technologies used in livestock metabolomics studies. These methods include, but are not limited to, infrared spectroscopy (FTIR), silicic acid column chromatography, immunoassays, the Kjeldahl method (for organic nitrogen measurement), ELISAs, and miscellaneous, lab-specific methods. Relative to other fields of metabolomics, livestock metabolomics appears to use NMR spectroscopy somewhat more and LC-MS somewhat less. This may simply reflect the availability of instrumentation or the preferences of major research groups in livestock metabolomics. Certainly, sample abundance and supply is not a significant issue in livestock metabolomics so, the use of tools that require higher-volumes, but offer more quantitative results (such as NMR) is not unexpected. However, NMR is not the most sensitive technique and certainly if livestock metabolomics researchers wish to extend their coverage of the livestock metabolome, they will certainly need to makes use of more LC-MS methods.

Another gap that was noted in livestock metabolomics research is the near complete absence of ICP (inductively coupled plasma)-MS studies to measure metal ion levels in tissues and biofluids. Indeed, only 2 studies used ICP-MS, with the most complete characterization being conducted by Saleem et al. [52] who reported the identification and quantification of 20 metals in bovine ruminal fluid. The importance of metal ions as micronutrients for animal health and animal productivity cannot be underestimated [80; 81]. Therefore, it is surprising that so little metal ion data has been collected or analyzed in livestock metabolomic studies. It was also noted that the use of fluxomics [82] or the measurement of metabolite flux using stable isotopes is completely absent in livestock metabolomics studies. Fluxomics is particularly useful in understanding metabolic sinks and sources. It is also useful for assessing nutrition and metabolic efficiency—topics, which are obviously important in livestock research. However, to conduct metabolic flux analysis, isotopically labeled (^13^C or ^2^H) feed needs to be used. Given the size of most livestock animals (relative to rats and mice) and the need for significant quantities of expensive, isotopically labeled feed, fluxomic studies are likely too difficult and costly to perform [83]. Likewise, the use of imaging mass spectrometry or IMS (which is becoming very popular in human metabolomics studies) was completely absent in livestock studies. Imaging mass spectrometry is particularly useful for analyzing tissues and for understanding the metabolic changes that take place during tissue development or tissue transformation [84; 85].

A good metabolomics study should use more than one analytical platform, and ideally as many different (orthogonal) platforms as possible to broaden the metabolite coverage. In our analysis we found that 69% of the published studies used just 1 platform (either NMR, HPLC-UV, LC-MS, GC-MS or ICP-MS), 15% used 2 platforms and only 3% used 3 or more analytical platforms. The remaining 13% of studies used relatively non-conventional platforms or assays (immunoassays, FT-IR, etc.). The most comprehensive metabolomic analysis was a study that used 5 different platforms (NMR, HPLC-UV, LC-MS, GC-MS and ICP-MS) to characterize the bovine ruminal fluid metabolome [52]. Looking through the more recent studies, there is a general trend towards using more than one platform and a growing trend towards using LC-MS techniques over NMR methods. However, the surprisingly high number of livestock metabolomic studies that still use only a single platform also represents a significant issue that the field must remedy. Certainly the trend in human metabolomic studies is to use at least 2 and often 3 or more different analytical platforms [2].

Another gap that was identified from this literature analysis was the general lack of integration of other omics techniques (proteomics, transcriptomics or SNP measurements) with reported livestock metabolomic studies. Indeed, only 5 papers (3 bovine and 2 swine metabolomics studies) used metabolomics in conjunction with genomics or proteomics. One paper of note was an investigation that used genomics and metabolomics to evaluate RFI (residual feed intake) from cross breeds of dairy and beef cattle [66]. This group of researchers used metabolomics and phenotypic data to support their genomics investigations and identified two genes (*TP53* and *TGFB1*) that were strongly associated with cellular functions driving feed efficiency. In another study by Lu and colleagues [50], the effect of genetic polymorphisms on dairy milk characteristics was evaluated using a combination of metabolomics and proteomics. This paper identified alterations in triglyceride composition and reported changes in the milk metabolome and proteome of dairy cows with the K232A (lysine to alanine substitution) polymorphism in the well-studied *DGAT1* gene. Given the growing trend towards systems biology research and the more “holistic” interpretations of multi-omics data in other fields of life science, the near absence of multi-omics studies represents an important gap in livestock metabolomics (and omics) research.

### Trends and gaps in metabolite quantification

The majority of livestock metabolomics publications are non-quantitative or semi-quantitative (yielding relative quantification) while 28.18% of published studies provide fully (absolute) quantitative data. The metabolites tracked in this review were categorized in two main groups: 1) quantified and 2) non-quantified metabolites. Any metabolite that was associated with an absolutely quantified value (millimolar, micromolar, nanomolar, mg/mL, ug/mL, etc.) in a given sample type was placed in the quantified category. The non-quantified group consists of either metabolites with no quantified value or ones that have only relative quantification (i.e. reported as a fraction or a percentage). Over all livestock species and all sample types, we found a total of 404 quantified metabolites and 666 non-quantified. The majority of both quantified and non-quantified metabolites are lipids and lipid-like molecules. Temporal trends in metabolite quantification show that proportionally fewer livestock metabolomics papers are providing quantitative data. For instance, 69% of papers published prior to 1999 had quantitative data, while 34% of papers from 1999–2010 and just 21% from 2011–2015 generated quantitative metabolite data.

Overall, livestock metabolomics still has an impressive proportion (~28%) of publications that report absolute concentration values. In contrast, most other fields of metabolomics quantify metabolites far less frequently [72]. Nevertheless, the steady decline in the proportion of livestock papers providing quantitative metabolomic data is not a good sign. The importance of absolute quantification in metabolomics cannot be over-emphasized. As a branch of analytical chemistry focusing on small molecule characterization, there is more than 100 years of history and a plethora of tools, standards and protocols designed specifically for absolute metabolite quantification [79]. Absolute quantification allows facile comparisons of readings between animals, research staff, platforms, laboratories and countries. Acquiring quantified values also allows one to determine normal and abnormal ranges for disease diagnosis, prediction as well as other relevant production measures. Obtaining quantified data and recognizing normal physiological concentrations is also a requirement in biomarker discovery [86; 60]. Indeed, absolute quantification and the existence of normal and abnormal ranges is the foundation to the entire field of clinical chemistry. In livestock metabolomics, having a “normal” quantified range specific for each animal species or breed is critical for defining referential “healthy” conditions. Likewise, being able to quantify specific changes in an animal’s metabolome allows one to identify “abnormal” conditions such as overt disease, malnutrition, pregnancy difficulties, and most importantly subclinical conditions for which no obvious clinical indicators are visible [51; 87].

### Trends and gaps in metabolite coverage

Based on our analysis of the literature and the definition of a metabolite given earlier, the majority of livestock metabolomics studies report ≤50 metabolites (79% of the total selected metabolomics publications) while the other 21% report >50 metabolites. The largest number of metabolites (or features) reported in a single paper was 647 [51] covering multiple biofluids for bovine samples while, the fewest reported was 8 (in a variety of papers from all different livestock species). As with metabolite quantification, there is a trend for more recent livestock metabolomics papers to report a greater number of metabolites. For instance, papers published prior to 1999 averaged 29 metabolites per study, those from 1999–2010 averaged 44 metabolites per study while, papers from 2011–2015 averaged 63. Among the later publications, the recent bovine study conducted by Sun et al. [51] who investigated potential biomarkers of milk production and quality using GC-time-of-flight/MS analyses of rumen fluid, milk, serum and urine claimed to detect the highest number of metabolites (i.e., 647). However, careful reading of the manuscript shows that they only formally identified 123. The remaining “metabolites” were unidentified MS peaks or features. In ovine metabolomic studies, Parveen and colleagues [88] reported 168 out of 205 detected metabolites using GC-MS to investigate sheep plasma and feces. Clark et al. [89] reported 97 metabolites out of the 571 detected features in caprine serum using a combination of both GC-MS and LC-MS. In equine metabolomics, the highest number of metabolites identified was from a study conducted by Escalona and colleagues [53] with 102 metabolites identified via NMR analysis of plasma, urine and fecal water. A porcine metabolomics study by Metzler-Zebeli et al. [90] reported 104 out of 132 detected serum metabolites using LC-MS.

Overall, our analysis shows a total of 1070 non-redundant or unique metabolites have been detected and/or quantified in the livestock metabolomics literature. Bovine studies covered the majority of detected metabolites (i.e., 768 different compounds) over multiple sample types. Porcine and ovine studies have the next highest number of detected metabolites with 412 and 285 different metabolites, respectively. Caprine and equine studies reported 167 and 109 different metabolites, respectively. The most frequently detected metabolites with >100 separate entries for different animals, biofluids or conditions include: alanine (124 times), valine (112 times), isoleucine (105 times), glycine (101 times), and lactate (101 times). In addition, 26 other metabolites were reported 50–100 times. Metabolites reported more than once and <50 times add to 560 while, 479 metabolites are reported only once.

It is important to provide some context to these numbers, especially with regard to metabolome studies reported for other animal or model species. The estimated size of the mammalian metabolome is >100,000 molecules [36; 2] and the total number of metabolites so far reported and/or theoretically expected to be in the human metabolome or HMDB is just over 42,000 [78]. While the number of expected or theoretical metabolites is large, the actual number of experimentally identified (and/or quantified) metabolites is actually quite small. For instance, based on the HMDB, the number of experimentally identified metabolites in the human metabolome is 3821 [78], in the *E*. *coli* metabolome it is 891 [41] and in the yeast metabolome it is 625 [40]. Among the different livestock species, it is clear that the coverage of the bovine metabolome is quite extensive and is approaching or even exceeding that of other model organisms. However, there is an obvious gap in terms of the coverage of other livestock species with caprine and equine metabolomes being very poorly characterized. Much more work is needed on goat and horse metabolomes to bring them up to the level seen in the bovine metabolome.

### Trends and gaps in animal breeds

While we have largely focused on examining metabolomics data for different livestock species, we also noticed some interesting trends with regard to the choice of specific breeds in each livestock species. Similar to other fields of bovine research, the majority (45%) of bovine metabolomic studies use either pure- or cross-bred Holsteins. A smaller amount (11%) of other studies used cross breeds to investigate various aspects of the bovine metabolome. Other common bovine breeds used in metabolomic studies include Charolais (7%) and Jersey (3%). In ovine metabolomic studies, the main breed reported is Suffolk (19%) while other breeds (i.e., Sarda) are reported only once or twice. For caprine metabolomics studies, the preferred breeds have been Norwegian (22%) with other breeds such as Saanen and Alpine being reported only once. Likewise, among equine and porcine metabolomic studies, Standardbred horses (33%) and Landrace sows (22%) were most frequently used. Interestingly, no breed information was provided in 18%, 33%, 22%, 17%, and 9% of the bovine, ovine, caprine, equine, and porcine metabolomics manuscripts, respectively. It is surprising that this essential information is not provided in the manuscripts. This suggests the reporting standards found in livestock metabolomics manuscripts still needs improvement.

Based on the above statistics, one of the more obvious gaps in current livestock metabolomics research is the limited variety of breeds being used in most metabolomic studies. The vast majority of the published research appears to be focused on just one or two main breeds i.e., Holstein in cattle, Suffolk in sheep, Standardbred in horses. Evidently, assessing breed differences and their potential impacts on the metabolome has not been a priority for most livestock researchers. However, it is important to remember that the existence of dozens of livestock breeds is a consequence of centuries of selection for very unique phenotypic qualities—some of which are likely determined by their metabolism or metabolome. Different breeds will be characterized by specific production or metabolic parameters and these may be fundamentally different between breeds. While the composition of mammalian (and livestock) metabolomes is likely to be highly similar, metabolite concentrations are expected to differ substantially between different breeds. Identifying the unique aspects affiliated with each breed’s metabolome is therefore, an important component of livestock metabolomics that should be considered in future studies. This is particularly true for purebred and breeding stock herds that are limited to very few animals/herds worldwide. Breeding stock animals provide most of the genetic background found in most commercial herds, which means they have a significant influence on the metabolome associated with their progeny. We were also surprised by the very limited research on the neonatal livestock metabolome. Indeed, we found only 16 neonate metabolomic studies, with 1 study focused on calves, 4 on lambs, 1 on kids, 10 on piglets and no studies on colts or foals.

### Trends and gaps in biomarker discovery

One of the strengths of metabolomics lies in its utility for biomarker discovery [91]. Because metabolites can be more easily, cheaply and routinely quantified than most other biological molecules, they are ideal for use in biomarker panels. Indeed, metabolite biomarkers continue to be developed and used in clinical applications at a much greater rate than genes or proteins [78]. In surveying the papers compiled for this review, we found a total of 11 livestock metabolomics papers that proposed candidate biomarkers. This included 5 papers in animal health, 1 in animal nutrition, 2 in animal production, 1 in animal reproduction and 2 for animal models of human health. These studies were limited to cattle, sheep and pigs with no metabolomic biomarker studies being reported for goats or horses. Of these papers, we observed that most reported fewer than 30 candidate biomarkers, with the lowest number being 2 [61]. A few reports used higher number of metabolites, i.e., 64, as part of a statistical model to increase the accuracy of prediction [92; 93]. The majority (55%) of metabolomic biomarker papers did not provide any quantitative data, but rather reported only relative metabolite trends (up or down relative to some indeterminate standard). This means that only 5 papers, all from the bovine group, effectively provided useful or verifiable biomarker data. Furthermore, only a single paper [20] reported follow-up validation studies where the initially discovered biomarkers were subsequently validated on a separate cohort of samples.

Based on our data, most biomarker studies were conducted with relatively small sample sizes with the majority of studies being done on fewer than 100 animals. The largest biomarker study was one conducted on 321 animals (1587 samples), which investigated prognostic biomarkers of ketosis in dairy cows using NMR spectroscopy [61]. Overall, the quality of biomarker studies done for livestock metabolomics is not particularly good, especially given the standards expected of human biomarker studies [91].

Nevertheless, among the reported biomarker studies, we did find some very interesting and compelling results. One example is a biomarker study of RFI and other feed efficiency traits in beef steers [20]. In this study, NMR spectroscopy was used to identify and quantify plasma metabolites associated with RFI, initially in a discovery population and subsequently in the validation cohort. Karisa et al. [20] reported 3 candidate biomarkers of RFI that significantly (P<0.05) account for >30% of the phenotypic variation for this trait. Other metabolites were proposed to be associated with average body weight, average feed intake, dry matter intake and average daily gain. In another interesting study, predictive biomarkers of transition diseases in dairy cows were investigated [21]. This study monitored only 12 dairy cows over four time points during the transition (pre- and post-calving) period. Blood samples were drawn to quantify the metabolome changes associated with various periparturient diseases post-calving. Using direct flow injection (DFI)-MS, Hailemariam and colleagues [21] profiled 120 blood metabolites of which 3 were suggested as candidate biomarkers for transition diseases, with a sensitivity and specificity of ≥85%. Another study reported by Gray et al. [94] looked into biomarkers associated with vaccine efficacy. Using UPLC-MS metabolomic measurement of plasma derived from Holstein male calves, Gray and colleagues [94] found 12 metabolites that were altered post-vaccination. These biomarkers are being proposed as a newer, more efficient route to optimise vaccination and to make vaccine formulation and benchmarking much more efficient and targeted. This paper emphasizes on the importance of disease prevention and vaccination procedures in livestock, especially in using new technologies such as metabolomics to enhance evaluation of vaccine efficacy.

Identification of biomarkers will not only improve disease diagnosis but also allow the opportunity for disease prediction prior to manifestation of clinical signs. For example, if a metabolic disorder can be predicted well before (sub)clinical manifestation, farmers can make informative decisions with regards to their management, feeding, housing, etc., to change the cascade of biological events leading to that disease. Predictive attempts of such can make a significant financial and sustainability difference by maintaining production quantity and quality, saving on costs associated with treatment, veterinary visits, preventing animal culling and thus, maintaining longevity.

### The livestock metabolome database

In assembling the material for this review, we identified a total of 1070 metabolites that have been detected and/or quantified in livestock metabolomic studies of cattle, sheep, goats, horses and pigs. This information has been systematically categorized into LMDB with all of the metabolites being fully described including information about the degree or quality of quantification (i.e., quantified, non-quantified) and the source sample types for each livestock species. All of the metabolites with quantitative data had their concentrations converted into a standardized concentration unit (i.e., μM) to improve consistency. In addition to the chemical data and source information, an abbreviated description of the experimental context for each metabolite was extracted from the articles and included in the online database (http://www.lmdb.ca). This information includes data on the analytical platform(s), experimental conditions, field of research, and animal breed used in acquiring the metabolomic data. All metabolites are linked to a standard HMDB (http://www.hmdb.ca/) identification number, which provides a freely-accessible and detailed description of the metabolite. A PubMed and/or DOI identifier is also associated with each metabolite entry, which provides a literature reference or a direct link to the article reporting that metabolite for readers who are interested in further details.

Only those metabolites that had reasonably complete descriptions (i.e., unique chemical names, sample types, source information, etc.) were included in the online database. A number of metabolites or “features” were identified during the review process but not included in the LMDB. These include those compounds that have either not been characterized at all (no chemical name, no data on sample types), or not fully characterized (unknown or undefined chemical structure). This collection of 415 “unknown” metabolites will be added to the LMDB once we can obtain sufficient structural and sample source information on them. Among the metabolites entered into the LMDB, 404 compounds were quantified and 666 were not. On a species level there were 768 bovine metabolites, 285 ovine metabolites, 167 caprine metabolites, 109 equine metabolites, and 412 porcine metabolites. Detailed descriptions of each compound are provided in the LMDB “metabocard” pages. Likewise, structural images, molecular formulas, names and synonyms, chemical classification/taxonomy information, physicochemical data (molecular weights, pI’s, pKa’s, boiling/melting points), referential spectral data (both experimental and theoretical NMR, MS/MS and EI-MS spectra), links to other online databases and full reference (authors, journals, volumes, etc.) information is also provided. The LMDB has been designed so that it can be easily browsed and it supports searches through standard text queries as well as via structure, mass, and spectral queries. Most of the information in the LMDB is hyperlinked to other resources within the LMDB, allowing for a more convenient and compact route to access the data. The LMDB is available at http://www.lmdb.ca. This database will be constantly updated with more metabolites and more detailed metabolite descriptions as more research in livestock metabolomics is published.

By assembling the LMDB and making this information freely available through both the web and this manuscript, we hoped to create a referential resource that other livestock researchers could readily use. Our past experience in assembling and maintaining the Human Metabolome Database (HMDB) clearly showed how useful a centralized, on-line resource could be in the field of human metabolomics [38]. Therefore, our expectation with the LMDB is that it will have a comparable impact on the field of livestock metabolomics. Indeed, we believe that establishing a comprehensive repository that stores and categorizes livestock metabolome information into a standardized format will be critical for future livestock research. It will also be important for identifying potential livestock disease biomarkers, improving animal selection (via metabolomic assays), enhancing animal nutrition and understanding novel biochemical mechanisms arising from various physiological perturbations. With more and more livestock metabolomics papers appearing each year and the continued growth in metabolite coverage, it will be challenging to maintain the LMDB. However, without even attempting to create the LMDB we suspect that livestock metabolomics would continue to lag behind the metabolomics activities seen in other areas (i.e. human, plant crops, microbes, food/beverage studies) and would face significant hurdles in the coming years trying to catch up.

## Conclusion

Metabolomics is less than 15 years old, yet it has already delivered some remarkable achievements. This includes significant improvements in the ability to identify many environmental contaminants and toxins [95], significant advances in food and nutrient characterization [7; 6], the identification of many novel biomarkers for disease risk including risk markers for diabetes [54], heart disease [96] and cancer [58] as well as promising leads for a variety of drugs and therapies [97]. Metabolomics is also well-positioned to provide some important advances in both livestock research and the livestock industry, especially as it relates to livestock health, breeding and production. A number of examples were highlighted in this review including metabolome discovery for normal metabolite composition and concentrations [52; 53], identification of biomarkers of transition diseases [21] as well as production traits in dairy [51] and beef cattle [20] with the goal of introducing prognostic strategies in animal health as well as increasing prediction accuracies. Our observations also showed that a wide variety of biofuids have received attention for metabolomics research such as metabolic profiling of milk, plasma, serum, and urine, minimizing animal welfare concerns.

However, in order for livestock metabolomics to deliver on the promise and the excitement seen in other areas of metabolomics research, it is important to carefully assess what has been accomplished, what is known and what still needs to be done. The intent of this review was to provide a critical overview of the trends and gaps in livestock metabolomics research. Specifically, we sought answers to 4 key questions: 1) What are the most common applications of metabolomics in animal science and where are they trending?, 2) What are the preferred metabolomics technologies livestock metabolomics and how are they evolving?, 3) What are the most obvious gaps or weaknesses in livestock metabolomics relative to other fields of metabolomics research? and 4) What are the known or measured metabolites for the 5 major livestock species (i.e., bovine, ovine, caprine, equine, and porcine) in different tissues and biofluids? In addressing the first 3 questions we focused on areas relating to: 1) Animal Choices; 2) Research Applications; 3) Sample Size; 4) Sample Type; 5) Instrumentation and Methodologies, 6) Quantification; 6) Metabolite Coverage; 7) Animal Breeds, and 8) Biomarker Identification. In many cases we were able to identify some clear trends while at the same time identifying important shortcoming or areas where further improvements could be made. It was apparent that livestock metabolomics appears to be ahead with regard to metabolite quantification, the diversity of research applications and its efforts in biomarker identification. On the other hand, it was also clear that livestock metabolomics (especially with regard to sample size, instrumentation and metabolite coverage) was lagging somewhat further behind than human, microbial or plant crop metabolomics.

Based on our assessment of the shortcomings with current livestock metabolomics studies, it is clear that future metabolomics research should focus on expanding or extending metabolome discovery using healthy control animals, increase sample numbers, direct more effort towards metabolite quantification, perform more integrated multi-omics experiments, use a greater variety of analytical platforms or techniques (including ICP-MS, MSI and fluxomics), increase the breadth of metabolite coverage (by using more sensitive platforms, such as ESI-MS), investigate a greater and new varieties of biosamples such as semen, amniotic fluid, saliva and urine, extend the number and types of animal breeds used in metabolomic studies and be more conscientious in the design and implementation of biomarker studies.

Another important outcome of this study was the collection and consolidation of livestock metabolite information into a single, centralized resource (the LMDB). It became readily apparent in conducting this review that the livestock metabolomics literature is highly diffuse and that valuable information is being “lost” or is not readily available. By compiling the LMDB and making an on-line version of the database freely available, we hope it could serve as a hub for livestock researchers and the livestock industry to further advance the field of livestock metabolomics.

## Supporting information

S1 Table PRISMA checklist. The preferred reporting items for systematic reviews and meta-analysis (PRISMA) checklist reflects 27 items under 7 main categories that highlights essential components of this systematic review.
(TIFF)
