# Supplementary figures and images for: Livestock metabolomics and the livestock metabolome: A systematic review
Source: PLoS One. 2017 May 22;12(5):e0177675. doi: 10.1371/journal.pone.0177675 (PMC5439675; doi:10.1371/journal.pone.0177675)

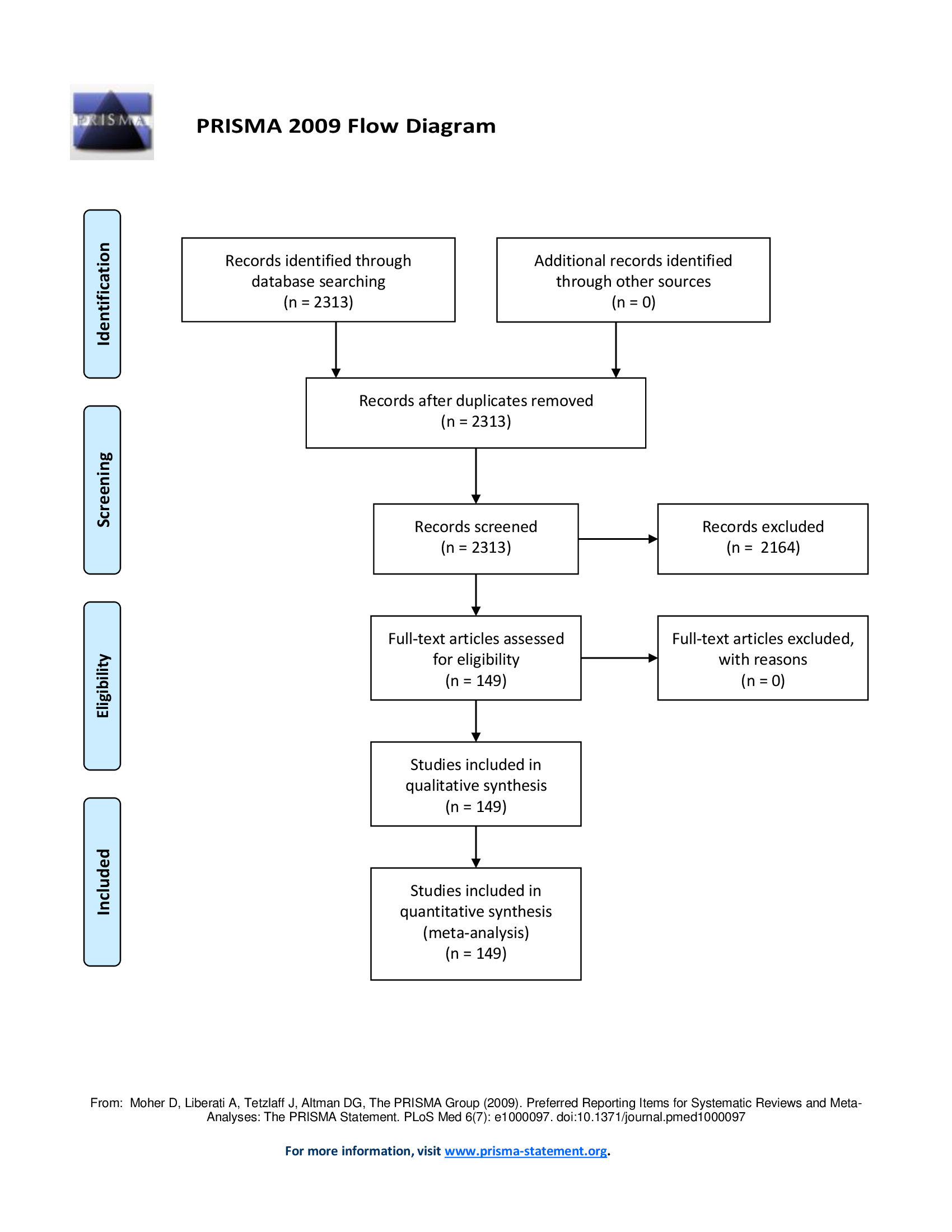

Supplement: S1 Table — The preferred reporting items for systematic reviews and meta-analysis (PRISMA) checklist reflects 27 items under 7 main categories that highlights essential components of this systematic review. (TIFF) [file pone.0177675.s001.tiff]
